# Cold and hot tumors: immunological determinants, cancer-immunity cycle dysregulation, and nanotechnology-driven therapeutic approaches

**DOI:** 10.1186/s43556-026-00534-0

**Published:** 2026-08-18

**Authors:** Mohammed S. Teiama, Asmaa Gohar, Mahmoud Amr, Mohamed El-Shinawi, Walid F. Elkhatib

**Affiliations:** 1 Department of Pharmaceutics and Industrial Pharmacy, Faculty of Pharmacy, Galala University, New Galala City, Suez, 43713 Egypt; 2 Department of Pharmaceutics and Industrial Pharmacy, Faculty of Pharmacy, Capital University (Former Helwan University), Cairo, 11795 Egypt; 3 Extract and Allergen Evaluation Lab, Egyptian Drug Authority (EDA), Giza, Egypt; 4 Department of Microbiology and Immunology, Faculty of Pharmacy, El Saleheya El Gadida University, El Saleheya El Gadida, 44813 Egypt; 5 Research and development formulation specialist at Al Andalous Pharmaceuticals, Giza, Egypt; 6 President of Galala University, New Galala City, Suez, 43713 Egypt; 7 Department of General Surgery, Faculty of Medicine, Ain Shams University, Cairo, 11566 Egypt; 8 Department of Microbiology and Immunology, Faculty of Pharmacy, Galala University, New Galala City, Suez, 43713 Egypt; 9 Microbiology and Immunology Department, Faculty of Pharmacy, Ain Shams University, African Union Organization St., Abbassia, Cairo, 11566 Egypt

**Keywords:** Cold tumor, Tumor microenvironment, Tumor antigen, Cancer immune cycle, Nanomedicine, Immunomodulators

## Abstract

Cancer remains a major global health burden and the second leading cause of mortality worldwide. Recent advances in cancer immunotherapy have emphasized the critical role of the tumor microenvironment (TME) in determining therapeutic outcomes, leading to the classification of tumors into immunologically “hot” and “cold” phenotypes. Cold tumors are characterized by low immunogenicity, limited immune cell infiltration, and a highly immunosuppressive microenvironment, resulting in poor prognosis and resistance to immune checkpoint inhibitors. Despite the development of multiple immunotherapeutic strategies, effective activation of antitumor immunity in cold tumors remains a major clinical challenge. Current approaches aim to initiate immune responses through priming strategies such as cancer vaccines and adoptive T-cell transfer, while simultaneously overcoming immunosuppressive signaling via immune checkpoint blockade. Additional strategies include depletion of myeloid-derived suppressor cells and enhancement of co-stimulatory pathways. However, these approaches are often limited by inefficient delivery, poor tumor penetration, and systemic toxicity. Nanotechnology has emerged as a promising platform for tumor microenvironment reprogramming. Nanocarriers enable targeted delivery of immunomodulatory agents, enhance antigen presentation, and improve immune activation while overcoming biological barriers such as dense stroma and abnormal vasculature. By integrating nanotechnology with immunotherapy, new opportunities arise to convert cold tumors into hot, immune-responsive phenotypes, thereby improving therapeutic efficacy and clinical outcomes.

## Introduction

Cancer remains a major global health challenge and continues to be one of the leading causes of mortality worldwide [1–3]. Despite significant advances in diagnosis and treatment, cancer progression is driven by several hallmark features, including sustained proliferation, resistance to apoptosis, angiogenesis, and metastasis, which collectively enhance tumor survival and adaptability [4–6]. In recent years, increasing attention has been directed toward the tumor microenvironment (TME), a complex and dynamic network composed of cancer cells, immune cells, stromal components, and extracellular matrix, which plays a critical role in regulating tumor progression, immune responses, and therapeutic outcomes [7–12]. The interaction between tumor cells and the immune system has led to a modern immunological classification of tumors into “hot” and “cold” phenotypes based on immune activity within the TME [13–15]. Hot tumors are characterized by high infiltration of cytotoxic cluster of differentiation (CD8⁺) T lymphocytes, elevated expression of inflammatory cytokines such as interferon-γ (IFN-_Ɣ_), and increased expression of immune checkpoint molecules, rendering them more responsive to immunotherapy [4, 16, 17]. In contrast, cold tumors exhibit limited immune cell infiltration, defective antigen presentation, and a strongly immunosuppressive microenvironment enriched with regulatory T cells, myeloid-derived suppressor cells, and cancer-associated fibroblasts, resulting in poor responsiveness to immune checkpoint inhibitors and unfavorable clinical outcomes [18, 19].

Although immunotherapy has revolutionized cancer treatment, its clinical efficacy remains inconsistent, particularly in patients with cold tumors [13, 20]. This limited responsiveness is largely attributed to the suppressive tumor microenvironment, impaired immune cell trafficking, and insufficient activation of antitumor immune responses [21, 22]. Consequently, converting cold tumors into hot, immune-responsive phenotypes has emerged as a central objective in cancer research, with growing interest in developing strategies that can modulate the tumor microenvironment, enhance antigen presentation, and restore effective immune surveillance [7, 14, 23].

This review provides an overview of the biological and immunological characteristics of cold and hot tumors, with a particular focus on the role of the tumor microenvironment and immune cell infiltration. It further discusses the dysregulation of the cancer-immunity cycle and highlights advanced therapeutic strategies including immunotherapy and nanotechnology-based approaches designed to reprogram the tumor microenvironment and promote cold-to-hot tumor conversion.

## Immunological determinants of cold and hot tumors

**Fig. 1:**
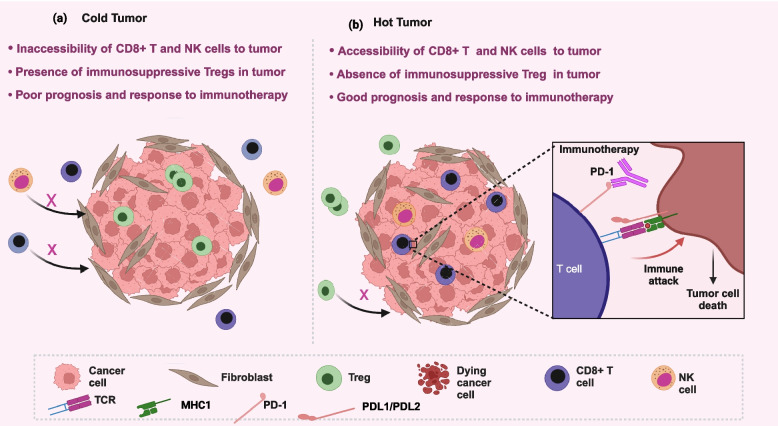
Cold tumor vs hot tumor: This figure represents cold tumor (**a**) and hot tumors (**b**). NK cells and CD8^+^ cells are excluded from cold tumor, but infiltered with immune suppressive immune cells (Tregs). **a** Cold tumors are characterized by poor prognosis and are not susceptible to immune therapy. On the contrary, **b** hot tumors are infiltered with CD8^+^ T cells and NK cells, but Tregs cell types are suppressed this improved prognosis and killing of tumor cells with immunotherapy treatment

The classification of tumors into immunologically hot and cold phenotypes is not defined by a single feature but reflects the integrated output of multiple, interacting immunological determinants operating within the tumor microenvironment (TME) (Fig. 1) [9, 13]. These determinants encompass the density and functional state of infiltrating lymphocytes, the antigenic and genomic properties of the malignant cells, the composition of immunosuppressive cell populations, the chemokine and cytokine milieu, and the stromal, vascular, and metabolic architecture of the tumor [14, 23]. Understanding how these factors collectively shape the immune landscape provides the conceptual framework for the dysregulation of the cancer-immunity cycle and for the therapeutic strategies discussed in the subsequent sections [24, 25].

### The immune contexture and phenotypic classification

The immune contexture defined by the type, density, location, and functional orientation of tumor-infiltrating immune cells is among the strongest determinants of both prognosis and response to immune checkpoint inhibitors (ICIs) [26, 27]. Hot, or T cell–inflamed, tumors are characterized by abundant intratumoral CD8⁺ cytotoxic T lymphocytes, a type I and type II interferon (IFN) transcriptional signature, and frequent adaptive expression of inhibitory checkpoints such as programmed death-ligand 1 (PD-L1), reflecting a pre-existing antitumor response that has been restrained rather than absent [16, 17]. Cold, or non-inflamed, tumors lack this productive effector infiltrate and are further subdivided into two mechanistically distinct patterns: the immune-excluded phenotype, in which T cells accumulate at the invasive margin but fail to penetrate the tumor bed, and the immune-desert phenotype, in which adaptive immune cells are essentially absent [13, 15]. This distinction is clinically consequential, as inflamed tumors such as melanoma and mismatch repair–deficient colorectal cancer are generally responsive to checkpoint blockade, whereas excluded and desert tumors such as pancreatic ductal adenocarcinoma and prostate cancer remain largely resistant [18, 28, 29].

### Tumor-intrinsic and genomic determinants

The most upstream determinants of tumor immunogenicity are intrinsic to the malignant cell. Tumor mutational burden (TMB) and the resulting neoantigen load provide the antigenic substrate for T-cell recognition; malignancies with high mutational rates, including melanoma, smoking-associated non-small cell lung cancer, and mismatch repair–deficient tumors, are enriched among the inflamed, checkpoint-responsive phenotype [16, 30]. High antigenicity is necessary but not sufficient, however, since the neoantigen repertoire must also be effectively processed and displayed. Consistent with this, Spranger and colleagues demonstrated that comparable neoantigen loads in T cell–inflamed and non-inflamed tumors do not elicit equivalent immune responses, indicating that additional determinants govern the phenotype [31].

Beyond antigenicity, oncogenic signaling actively sculpts the immune microenvironment. Tumor-intrinsic activation of the WNT/β-catenin pathway suppresses the chemokines required to recruit BATF3-lineage conventional dendritic cells (cDC1), producing a non-inflamed TME; loss of the tumor suppressor (PTEN), with consequent Phosphatidylinositol 3-Kinase/Protein kinase B (PI3K–AKT) activation, similarly diminishes T-cell infiltration; and loss-of-function of STK11/LKB1 defines an immunologically cold, checkpoint-resistant subset in lung cancer. Oncogenic MYC signaling upregulates CD47 and PD-L1, further promoting immune evasion. Finally, integrity of the IFN-γ–JAK–STAT axis is essential, since defects in this pathway blunt major histocompatibility complex (MHC) upregulation and chemokine induction, rendering tumors functionally invisible to an ongoing immune response [16, 17].

### The immunosuppressive cellular landscape

Even when antigen and effector cells are present, the balance between cytotoxic effectors and immunosuppressive populations determines whether an antitumor response can proceed. Cold tumors are typically enriched for regulatory T cells (Tregs), myeloid-derived suppressor cells (MDSCs), and tumor-associated macrophages polarized toward an immunosuppressive M2-like phenotype, which collectively suppress effector T-cell function through transforming growth factor beta (TGF-β), Interleukin-10 (IL-10), arginase, inducible nitric oxide synthase, and checkpoint-ligand expression [19, 32]. Dysfunction or paucity of cDC1s impairs antigen cross-presentation and effector priming, while reduced numbers or activity of natural killer (NK) cells compromise innate surveillance — a determinant of particular importance in tumors that downregulate MHC class I to escape CD8⁺ recognition [9, 23]. In hot tumors, by contrast, the effector-to-suppressor balance favors productive cytotoxicity.

### Chemokine, cytokine, and interferon networks

The recruitment and retention of effector T cells within the tumor is governed by a defined chemokine (CK) program. The CXCR3 ligands CXCL9, CXCL10, and CXCL11 — induced downstream of IFN-γ and produced largely by cDC1s — are the principal mediators of effector T-cell trafficking, and their expression closely tracks with the inflamed phenotype and with clinical benefit from ICIs [16, 17]. Cytosolic tumor-derived DNA further amplifies this circuit through the cGAS–STING pathway, driving type I IFN-﻿γ production that licenses cDC1 recruitment and antigen cross-presentation. In cold tumors, this circuit is disrupted, and the milieu is instead dominated by immunosuppressive mediators such as TGF-β, IL-10, and vascular endothelial growth factor (VEGF), and by cancer associated fibroblasts (CAF-derived CXCL12), which misdirects cytotoxic T cells away from the tumor nest [21, 22]. The self-reinforcing sequence, in which IFN signaling drives chemokine production that in turn recruits effector T cells, therefore represents the molecular hallmark distinguishing hot from cold tumors [13, 16].

### Stromal, vascular, and metabolic determinants

The immune-excluded phenotype is frequently imposed by non-cellular barriers. Cancer-associated fibroblasts (CAFs) and a dense, collagen-rich extracellular matrix physically restrain T cells at the tumor margin, a process driven largely by TGF-β signaling [21, 22, 28, 33]. Aberrant, disorganized tumor vasculature, sustained by VEGF, impairs lymphocyte adhesion and extravasation through downregulation of intracellular adhesion molecules (ICAM-1) and vascular cell adhesion molecule 1 (VCAM-1) while favoring immunosuppressive myeloid recruitment [34]. These structural barriers are reinforced by a hostile metabolic environment: hypoxia promotes immunosuppressive myeloid polarization and angiogenesis via hypoxia inducible factor (HIF-1α); extracellular adenosine generated by the ectonucleotidases CD39 and CD73 suppresses T-cell effector function; and tryptophan catabolism by indoleamine 2,3-dioxygenase (IDO), together with lactate accumulation and arginine depletion, further impairs lymphocyte proliferation and cytotoxicity [32, 35, 36].

Collectively, these tumor-intrinsic, cellular, chemokine, stromal, vascular, and metabolic determinants define where a given tumor lies along the hot-to-cold spectrum and dictate whether the successive steps of the cancer-immunity cycle can proceed efficiently. The following section examines how these determinants translate into dysregulation of the cancer-immunity cycle in cold tumors [13, 23, 37].

## Cancer immunity cycle

**Fig. 2:**
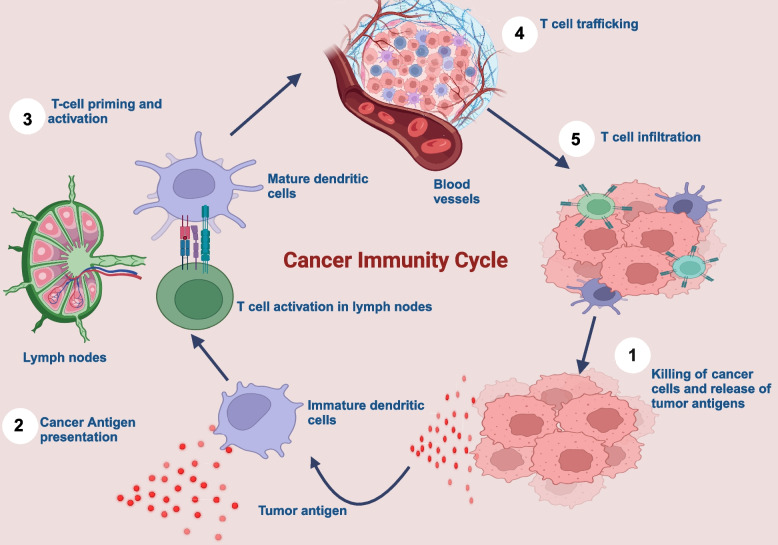
Cancer immunity cycle: Cancer immunity cycle starts with tumor antigen release that presented on to immature dendritic cells. (step 1, 2). In step 3, T cell are activated in lymph nodes triggering its migration through blood vessels and then infiltered into tumor stroma causing tumor cells killing and finally tumor antigen release triggering immunity cycle repetition

Cancer immunity cycle (Fig. 2) is a series of functional immunological events needed to obtain an effective antitumor immune response. The process is initiated by releasing neoantigens from the killed tumor cells as a first step. The second step starts by presenting of the released tumor antigens via MHC class I (MHC I) and MHC class II (MHC II) molecules onto cell surface. During this step, the released tumor antigens are processed into antigenic epitopes by antigen-presenting cells (APCs), like dendritic cells (DCs). In the third step, the activation and proliferation of tumor-specific CD4^+^ and CD8^+^ T cells occur through the recognition of epitopes by T cell receptors (TCRs) on T cells in lymphoid tissues. Finally, T cells are infiltered into tumor cells via blood vessels during the fourth and fifth steps causing cytotoxic tumor killing, antigen release and the cancer-immunity cycle are repeated again supporting anti-tumor activity [37].

The functionality of the cancer-immunity cycle varies significantly between hot and cold tumors, reflecting differences in antigen availability, immune activation, and tumor microenvironment composition. These variations ultimately determine the effectiveness of antitumor immune responses and responsiveness to immunotherapy.

### Step 1: Antigen release

Hot tumors are characterized by enhanced tumor antigen and neoantigen release, often due to high tumor mutational burden and ongoing immune-mediated cytotoxicity. This continuous release sustains immune recognition and amplifies downstream immune responses. In contrast, cold tumors typically exhibit low antigenicity, either due to low mutational burden or non-immunogenic forms of cell death, resulting in insufficient initiation of the cancer-immunity cycle. For example, Melanoma and smoking-associated non-small cell lung cancer (NSCLC) display high neoantigen load (hot tumors), whereas pancreatic cancer and prostate cancer often show low antigen release (cold tumors) [13, 38].

### Step 2: Antigen presentation

In hot tumors, antigen-presenting cells, particularly dendritic cells, efficiently process and present tumor-derived antigens via MHC class I and II molecules, enabling effective priming of T cells. Conversely, cold tumors frequently exhibit impaired antigen presentation due to dendritic cell dysfunction, reduced MHC expression, or defective antigen processing machinery. For example, MSI-high colorectal cancer demonstrates robust antigen presentation, whereas many solid tumors such as glioblastoma exhibit defective antigen presentation pathways [39, 40].

### Step 3: T cell priming and activation

Hot tumors support strong activation and proliferation of tumor-specific CD4⁺ and CD8⁺ T cells in lymphoid organs, driven by effective antigen presentation and co-stimulatory signaling. In contrast, cold tumors display inadequate T cell priming, often accompanied by an immunosuppressive dominance of regulatory T cells (Tregs) and inhibitory cytokines. For example, Renal cell carcinoma and melanoma show strong T-cell priming, while ovarian cancer often exhibits impaired T-cell activation [41].

### Step 4 and 5: T cell trafficking and infiltration

A hallmark of hot tumors is the efficient trafficking and infiltration of activated T cells into the tumor microenvironment, mediated by chemokines such as CXCL9 and CXCL10. In cold tumors, however, T cell trafficking is impaired due to abnormal vasculature, dense extracellular matrix, and stromal barriers, which physically and functionally exclude immune cells. For example, Inflamed tumors such as melanoma show high CD8⁺ T cell infiltration, whereas pancreatic ductal adenocarcinoma (PDAC) exhibits strong stromal exclusion of T cells [42, 43].

### Step 6 and 7: Tumor recognition and killing

In hot tumors, infiltrating cytotoxic T lymphocytes effectively recognize tumor cells via antigen-MHC complexes and induce tumor cell apoptosis, thereby reinforcing the cancer-immunity cycle through further antigen release. In contrast, cold tumors evade immune destruction through multiple mechanisms, including immune checkpoint overexpression, accumulation of myeloid-derived suppressor cells (MDSCs), and cancer-associated fibroblasts (CAFs), leading to ineffective tumor killing. For example, Checkpoint blockade therapies (e.g., anti-PD-1) are highly effective in melanoma (hot tumor), but show limited efficacy in prostate cancer (cold tumor) [20, 44].

This framework was first outlined by Mellman in 2013 [24], who described the traversal of blood-derived T cells through the stromal barrier in route to the tumor core [25]. The immune system proceeds through a series of stepwise, iterative events that must occur in this sequence to achieve effective killing of cancer cells. T The first step involves the release of neoantigens, which are captured by DCs and activate anticancer T-cell responses; this activation is typically accompanied by immunogenic signals such as proinflammatory cytokines and factors released by dying cancer cells or the gut microbiota. In the second step, DCs present the captured antigens on MHC molecules to T cells, marking the starting point of the third step, in which effector T-cell responses against cancer-specific antigens are primed. This third step is the most critical and rate-limiting stage of the cycle: the ratio and balance between effector and regulatory T cells, which determines the nature of the immune response, is established here and represents the key determinant of the eventual outcome. The fourth through seventh steps then proceed in sequence, beginning with trafficking of activated effector T cells into the tumor core, where they engage the T-cell receptor on the surface of cancer cells. Effector T cells then recognize the cognate antigen bound to MHC I, killing the targeted cancer cells at the final step [24]. This killing step is what drives repetition of the cycle, releasing additional tumor-associated antigens (restarting step 1) and reinforcing the immune response in subsequent cycles. In cancer patients, however, this cycle often fails to function properly: the TME suppresses effector T cells, tumor antigens go undetected, regulatory T cells are generated in place of effector T cells, and T-cell infiltration into the tumor core is blocked [19].

## Comparative study for hot and cold tumor

Hot and cold tumors behave completely different in regarding the immune cell profiles, biomarker expansion, and response to the immunotherapies. The following table include a simplified comparison to explain the difference in behavior (Table 1) [4, 45–48].

**Table 1:** The difference in behavior between hot tumor and cold tumor

Feature	Hot Tumor	Cold tumor
Immune cell infiltration	High infiltration of CD8⁺ T cells, NK cells, dendritic cells, presence of tertiary lymphoid structures (TLS)	Low or absent T-cell infiltration, immune-desert or immune-excluded phenotypes
Tumor microenvironment TME	Pro-inflammatory; enriched with IFN-γ, chemokines ligand (CXCL9, CXCL10, CCL5)	Immunosuppressive; high Tregs, MDSCs, M2 macrophages, suppressive cytokines
Antigen presentation	Efficient antigen presentation; intact MHC expression	Impaired antigen presentation; defective MHC machinery
Tumor mutational burden (TMB)	High TMB, increased neoantigens	Low TMB, reduced immunogenicity
Immune checkpoint expression	High PD-1/PD-L1 expression indicating active immune engagement	Low or heterogeneous checkpoint expression
Stromal barriers	Less restrictive stroma allowing immune infiltration	Dense stroma and abnormal vasculature limiting immune penetration
Metabolic environment	Supports immune cell function	Nutrient depletion, high lactate → T-cell dysfunction
Cytokines profile	Pro-inflammatory cytokines dominate	Anti-inflammatory and suppressive cytokines dominate
Response to immunotherapy	High response to immune checkpoint inhibitors (ICIs)	Poor response; primary resistance to ICIs
Examples	Melanoma, NSCLC, renal cell carcinoma	Pancreatic, Prostatic, and colorectal cancers

Hot (immune-inflamed) tumors are characterized by a highly active immune microenvironment with well-defined molecular and biomarker signatures of ongoing antitumor immunity. These tumors typically exhibit upregulated PD-L1 expression driven by interferon-gamma (IFN-γ) signaling, reflecting adaptive immune resistance mechanisms [13, 41]. They are also associated with high tumor mutational burden (TMB), which increases neoantigen load and enhances T-cell recognition [47]. At the cytokine level, hot tumors display a Th1-skewed inflammatory profile, including IFN-γ and chemokines such as CXCL9 and CXCL10 that facilitate recruitment and activation of CD8⁺ cytotoxic T lymphocytes [13]. Importantly, the antigen presentation machinery is functionally intact, with preserved MHC class I expression and efficient dendritic cell priming, enabling effective initiation of adaptive immune responses. Collectively, these features underlie the strong responsiveness of hot tumors to immune checkpoint blockade therapies [13, 49].

Cold (immune-desert or immune-excluded) tumors, in contrast, lack effective immune activation and display molecular features associated with immune evasion. These tumors often show low or heterogeneous PD-L1 expression and are typically linked to low TMB**,** resulting in limited neoantigen availability and poor immunogenicity [13, 47]. Their cytokine milieu is dominated by immunosuppressive pathways**,** particularly transforming growth factor-beta (TGF-β) signaling and anti-inflammatory cytokines such as IL-10, which inhibit T-cell infiltration and function. Furthermore, cold tumors frequently exhibit defective antigen presentation, including downregulation of MHC class I molecules and impaired dendritic cell activity, leading to insufficient T-cell priming [9, 50]. These molecular and immunological deficiencies contribute to intrinsic resistance to immune checkpoint inhibitors and define the major challenge in converting cold tumors into immunologically active (hot) phenotypes [13, 18, 49].

While, the cancer immunity cycle provides a mechanistic framework for immune activation, several barriers can disrupt this process and sustain the cold tumor phenotype. The following section discusses the main challenges that hinder immune activation and limit the efficacy of current immunotherapies.

## Challenges in treating cold tumors

Cold tumors show different defensing mechanism due to the presence of tumor antigen, failure of homing to the tumor bed, lack of T-cell stimulation, and disorder in the presentation and processing of tumor antigen.

### Absence of tumor antigens

**Fig. 3:**
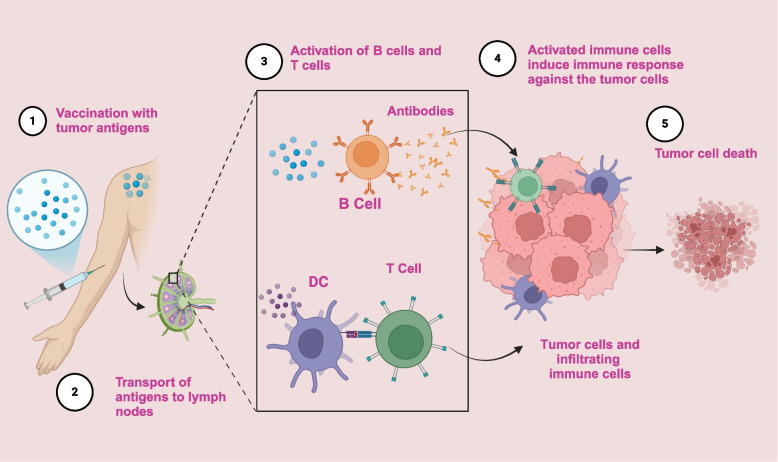
Vaccination with tumor antigens: DCs uptake and process tumor antigens after administration of the vaccine. They are cross-presented to MHC II or MHC I. Immune cells are activated after antigen-loaded DCs migration to lymph nodes. Activated B cells and T cells activate tumor death programme (apoptosis) through ADCC, and the latter proliferate and differentiate into memory T cells and effector T cells. Activated immune cells infiltered tumor cells and induce an anti-tumor immune response

The cancer vaccination is achieved by loading of tumor antigens (TAs) on a specific carrier to directly stimulate the immune system via spurring of the host immune response against cancerous cells. TAs should be immunogenic through the activation of B/T cell responses, presented on the cancer cell surface via initiating of histocompatibility complex (MHC), and highly selective to the type of the targeted cancer to be highly expressed on tumor cells (Fig. 3) [51, 52]. The first reported application of TA was in human melanoma, whereas Melanoma associated antigen 1 (MAGE-1) tumor antigen was recognized by the cytolytic T lymphocyte [53].

**Fig. 4:**
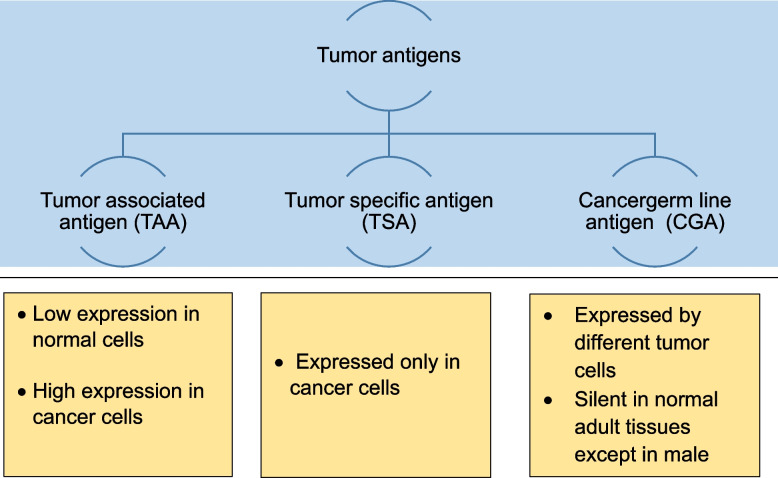
Tumor antigens classes: Tumor antigens are classified into three antigens expressed in cancer cells except CGA is expressed in cancer cells and male germline and trophoblastic cell

There are three categories of tumor antigens: the first category is the tumor associated antigen (TAA), or tumor-associated antigens. The second and third categories are tumor specific antigen (TSA), or tumor-specific antigens, and CGA, or cancer germline antigens (Fig. 4) [54]. TAA exhibits an elevated appearance in cancer cells and a declining presence in normal cells [55]. On the other hand, TSA is expressed by tumor cells only and plays an essential turn in the recognition process by CD8 T cells [56]. CGA exhibits different behaviors whereas it was found to be expressed by a number of different tumor cells and totally silent in normal adult tissues except in male germline and trophoblastic cells [57]. Tumors are believed to have a high number of TSA and CGA; however, this doesn’t seem to be the case. It was found that the presence of the tumor antigens doesn’t represent the main or only limiting factor in this process. A study by Spranger et al. revealed that the presence of a comparable quantity of tumor antigens in non-T cell-inflamed and T cell-inflamed solid tumors did not exhibit a similar immune response, indicating that there are other factors and mechanisms affecting the lack of T cell priming [31].

Tumor associated antigens are classified into several categories according to the pattern of expression in normal tissues. The first type is the overexpressed antigens which exhibit an elevated expression levels in cancer cells and declined expression levels in normal cells. The examples that belong to this type: Survivin, livin, tumor protein (TP53), mesothelin, human telomerase reverse transcriptase (hTERT), and HER2/*neu* [51, 58–65]. The second type is the tissue differentiation antigens which characterized by the expression in tumor cells and the normal cells from which the tumor was initiated. The examples that belong to this type: prostate-specific antigen, tyrosinase, Gp100, mammaglobin-A, carcinoembryonic antigen, and Melan-A/Mart-1 [66–71]. While the third common type is known as cancer testis antigens or tumor germline antigen as it expressed with high level at germline cells of the ovaries, placenta and testis. This type include New York esophageal squamous cell carcinoma (NY-ESO-1), synovial sarcoma X breakpoint 2 (SSX-2), melanoma associated antigen (MAGE), as well as B melanoma antigen (BAGE) and G melanoma antigen (GAGE) [53, 72–75].

The loss or defective processing of tumor antigens therefore represents a major obstacle to effective T-cell priming, contributing directly to the cold tumor phenotype and reduced immunotherapy responsiveness.

### Lack of T cell activation

The second step in immune anti-tumor response is presenting tumor antigen on dendritic cells (DC) to prime and activate several T cells. Several DC subtypes exist, including type 1 conventional DCs (cDC1s) and type 2 conventional DCs (cDC2s), with cDC1s being particularly effective at antitumor responses. Evidence suggests that a higher ratio of cDC1s to monocytes favors a protective antitumor immune response. Several murine studies have shown that rejection of transplanted tumors depends on the presence of cDC1s, confirming their role in protective antitumor immunity. Immune activation proceeds as a chain reaction, in which each step triggers the next. Mature, activated antigen-presenting DCs are essential for T-cell activation. APCs are activated by pathogen-associated molecular patterns (PAMPs) or danger-associated molecular patterns (DAMPs) via pattern recognition receptors (PRRs), which in turn prime and activate naive T cells. In turn DCs activates NK-κB, production of chemokines, induction of co-stimulatory molecules, and cross priming [76]. In the absence of DAMPs, DCs remain immature and instead produce immunosuppressive mediators such as TGF-β (transforming growth factor). Recent studies have also shown that DCs lacking or expressing a variant of Formyl Peptide Receptor 1 (FRP1) exhibit failure of T-cell activation and a poor immune response in tumor types such as colon and breast cancer [77]. Failure to activate CD40 via helper CD4^+^ T cells leads to failure of CD8^+^ T-cell activation, as well as impaired cytokine production and cross-priming of exogenous antigens [78].

### Deficient homing of immune cells to tumor sites

Recent studies have shown a marked reduction in CCL4 production in certain tumors, such as β-catenin-positive melanoma, accompanied by a crossponding decline in the recruitment of BATF3 DCs to the tumor site [42]. As a result, CTLs fail to migrate to the tumor site because of the resulting deficiency of CXCL9 and CXCL10, which are normally produced by BATF3 DCs [79]. Furthermore, it was found that direct injection of BATF3 DCs into the tumor site has been shown to produce modest tumor suppression improved T-cell infiltration in β-catenin-positive tumors [80]. Separately, activation of the PI3K/AKT pathway following loss of the tumor suppressor gene PTEN reduces lipidation of the autophagosome protein light-chain 3 (LC3). This decreases autophagic activity, impairing T-cell-mediated priming of the antitumor response [81].

Reticular activating system (RAS), a gene related to tumor improvement, can lead to enhancement of multiple tumorigenesis pathways such as mitogen-activated protein kinase (MAPK) and PI3K [40]. Furthermore, oncogenic signaling through MYC increases the expression of CD47 and PD-L1 on tumor cells. CD47 binds the inhibitory receptor signal-regulatory protein-α (SIRPα) on the surface of APCs such as macrophages and DCs, inhibiting phagocytosis of tumor cells and interfering with antigen uptake [82, 83].

The interaction between chemokines and their receptors on effector T cells plays a role in the trafficking of effector T cells to the site of tumor. These chemokines include CCL4, CCL5, CXCL9, CXCL10, CXCL16, and CX3CL1, and their reduced production has been reported to cause T-cell exclusion [84, 85]. For instance, BATF3 DCs are the major sources of CXCL9 and CXCL10, and the absence of BATF3 DCs results in low CXCL9 and CXCL10 expression; epigenetic regulation of the corresponding genes further maintains this low expression in tumor cells. This has been confirmed in several studies: in ovarian cancer, suppression of CXCL9 and CXCL10 is mediated by DNA methylation and histone lysine methylation, controlled by DNA methyltransferases (DNMTi) and the enhancer of zeste homolog 2 (EZH2). CXCL12, produced mainly by CAFs, plays an additional role by misdirecting CTLs away from the tumor and toward extra-tumoral tissue [86].

T cell infiltration is regulated not only by chemokines but also by the vasculature; for T cells to enter the tumor site, several sequential steps must occur. They must first enter the tumor circulation, then adhere to endothelial cells, and finally migrate across the vessel wall [24]. This migration depends on intercellular adhesion molecules (ICAMs) and vascular adhesion molecules VCAMs [86, 87], and downregulation of these molecules reduces T-cell migration into the tumor [87]. Moreover, vascular endothelial growth factor (VEGF) promotes the angiogenesis process through formation of new vessel [88].

### Defect in tumor antigen processing and presentation

Another major tumor evasion mechanism is the down regulating MHC I [89]. Low MHC I expression varies even within a single cancer type; the cancers most commonly affected include breast, prostate, colorectal, non-small cell lung (NSCLC), and hepatocellular carcinoma [90]. Any defect in the MHC I pathway will lead to a reduction or uneven expression of MHC I such as loss of the MHC I heavy chain, immunoproteasome subunit, Tapasin, ß2-microglobulin, and human endoplasmic reticulum aminopeptidase (ERAP1) [91]. Once MHC I is lost or reduced on the surface of the antigen, the immune system, specifically CD8^+^, cannot detect the presence of the tumor [92]. While cold tumors have different mechanisms of defense, there are different ways of overcoming such obstacles. Some of which are: 1**)** oncolytic viruses; 2) stromal targeting; 3**)** cancer vaccines; and 4) adoptive cell transfer [93].

## Advanced strategies to convert cold tumors into hot tumors

The transformation of cold tumors into hot, immune-responsive phenotypes requires coordinated therapeutic interventions targeting multiple steps of the cancer-immunity cycle. Cold tumors are characterized by impaired antigen presentation, defective T-cell activation, restricted immune infiltration, and a highly suppressive tumor microenvironment (TME). Therefore, effective strategies must act synergistically to initiate immune responses, enhance T-cell function, remodel the TME, and support sustained antitumor activity [14, 43, 45].

### Initiating the cancer-immunity cycle

**Fig. 5:**
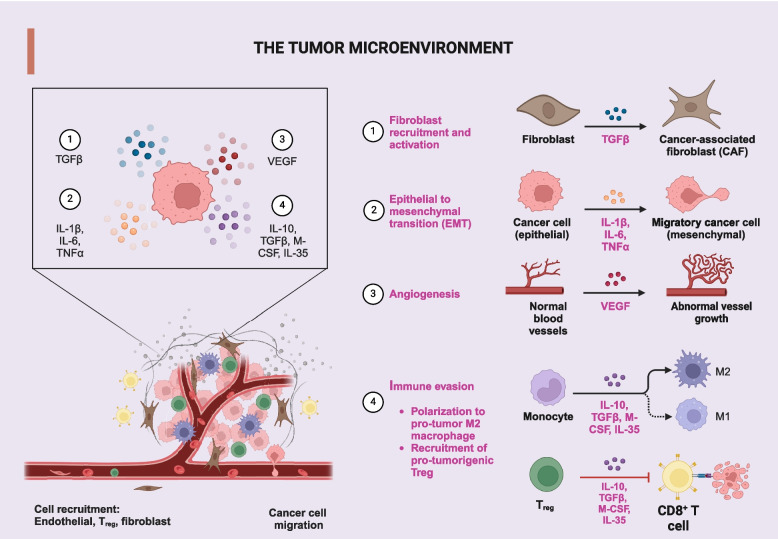
Tumor micronenvironoemnt: Cancer cells release molecules such as TGFß, IL1ß, IL-6, TNF-α, VEGF, IL-10, IL-35, and M-CSF that modify the tumor microenvironment and contribute to cancer proliferation and growth through immune evasion, angiogenesis, and metastatic niche formation. The tumor microenvironment (TME) consists of cellular and acellular components. The cellular components locate surrounding the tumor mass such as, immune cells, epithelial cells, and fibroblasts, while, the acellular components include the blood vessels and extracellular matrix

Current cancer vaccines are designed to induce immune responses against tumor cells by enhancing antigen recognition and T-cell activation. Among the FDA-approved vaccines, Bacillus Calmette–Guérin (BCG), derived from *Mycobacterium bovis*, is widely used for the treatment of non–muscle-invasive bladder cancer. Although its exact mechanism remains incompletely understood, BCG is administered intravesically following tumor resection and is known to stimulate local immune activation. Another approved vaccine, Sipuleucel-T, is used for castration-resistant prostate cancer and is generated by ex vivo incubation of patient-derived antigen-presenting cells (APCs) with a recombinant antigen fused to prostatic acid phosphatase (PAP) and granulocyte–macrophage colony-stimulating factor (GM-CSF) **(**Fig. 5**)**, thereby enhancing antigen presentation and T-cell priming. Despite their ability to induce immune responses, cancer vaccines often demonstrate limited efficacy as monotherapies, highlighting the need for combination approaches [94–96].

**Fig. 6:**
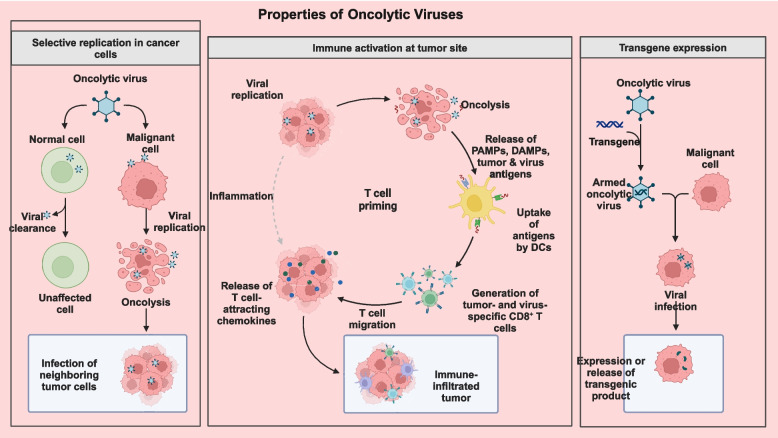
Properties of Oncolytic viruses: Oncolytic viruses (OVs) exhibit significant replication with noticed selectivity in the malignant cells and clear absence in the normal cells due to viral clearance. The replication process starts with tumor cell lysis and the release of virus progeny. Additionally, oncolysis induces the release of antigens, leading to the priming of T cells. Inflammation attracts T cells to the tumor. Further, OVs can be modified to encode transgenes, confirming their specific delivery to the tumor microenvironment in addition to activating an antitumor immune response

In this context, initiation of the cancer-immunity cycle represents a key strategy for overcoming the cold tumor phenotype. Oncolytic viruses play a central role in this process, as they selectively infect and replicate within tumor cells, leading to tumor cell lysis and the release of tumor-associated antigens (Fig. 6). This promotes dendritic cell (DC) activation and enhances T-cell priming. Furthermore, oncolytic viruses can be engineered to express immunostimulatory molecules, thereby amplifying antitumor immune responses.

A representative example is Talimogene laherparepvec (T-VEC), a genetically modified herpes simplex virus in which the infected cell protein (ICP34.5) gene, responsible for viral pathogenicity, and ICP47, associated with immune evasion, have been deleted to improve safety and immune recognition. In addition, T-VEC is engineered to express GM-CSF, which enhances the recruitment and activation of dendritic cells and other immune effector cells. This dual mechanism—tumor cell lysis and immune stimulation—facilitates both antigen release and effective T-cell priming [97–99].

Clinically, T-VEC has been approved by the FDA and has demonstrated both local and systemic antitumor effects, including responses in non-injected metastatic lesions. Moreover, its combination with immune checkpoint inhibitors has shown enhanced therapeutic efficacy. Similarly, other vaccine strategies, such as GVAX, have demonstrated increased T-cell infiltration in pancreatic cancer models; however, the presence of non-responders suggests that vaccine-based approaches alone are insufficient, reinforcing the importance of combination therapies for effective cold-to-hot tumor conversion [97, 100–102].

### Enhancing T-cell activation and function

Adoptive T-cell therapies (ACT), including chimeric antigen receptor (CAR-T) cells and tumor-infiltrating lymphocytes (TILs), represent powerful strategies for directly enhancing antitumor immunity. These approaches involve the identification and isolation of tumor-reactive T cells, followed by their ex vivo expansion and reinfusion into the patient. Prior to infusion, patients typically undergo lymphodepletion to improve the engraftment, persistence, and functional activity of transferred T cells, thereby enhancing therapeutic efficacy. CAR-T cells are genetically engineered to express synthetic receptors composed of an extracellular antigen-recognition domain and intracellular signaling domains that activate T-cell responses. A major advantage of CAR-T therapy is its ability to recognize tumor antigens in an MHC-independent manner, allowing for effective targeting even in tumors with impaired antigen presentation. Notably, CD19-directed CAR-T cells have demonstrated remarkable success and are now considered a standard treatment for certain B-cell malignancies. In contrast, TIL-based therapy relies on the expansion of naturally occurring tumor-reactive T cells isolated from tumor tissues. These cells are often selected based on markers such as CD137, which are associated with enhanced tumor reactivity and interferon-γ production. Recent advancements, including gene-editing approaches such as zinc finger nucleases, have been explored to reduce inhibitory receptor expression (e.g., PD-1), thereby improving T-cell function and cytokine production [94, 97].

Despite their promise, both CAR-T and TIL therapies face challenges related to the immunosuppressive tumor microenvironment (TME), which can limit T-cell infiltration and function. Therefore, combining adoptive T-cell therapies with immune checkpoint inhibitors represents a promising strategy to overcome these limitations and enhance therapeutic outcomes. However, additional challenges, including high production costs, scalability, and complex manufacturing processes, remain significant barriers to the widespread clinical application of these advanced therapies [103].

### Remodeling the immunosuppressive tumor microenvironment

#### Stromal targeting approaches

The tumor stroma makes T -cells infiltration into the tumor cells challenging. This is due to the physiological nature of the TME, where the media is acidic and hypoxic. Several studies were established to disrupt the hyaluronic acid (HA) holding the stoma together by the means of pegvorhyaluronidase (PEGPH20) in addition to nabPacitaxel. The results were not satisfying. In another recent study, CXCR4 inhibitors were used along with anti PD1 therapy in PDAC metastatic patients [104]. The treatment decreased TME and density of MDSC and increased the number of infiltrating CD8^+^ cells [105].

**Fig. 7:**
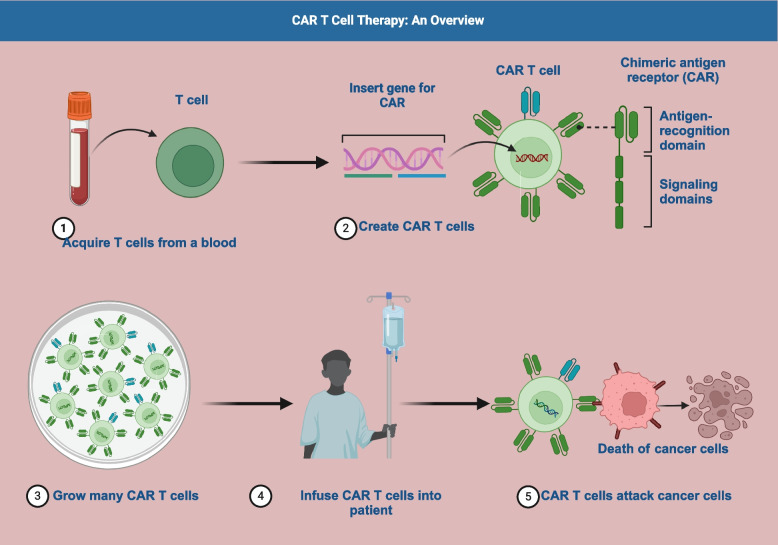
CAR T Cell therapy: an overview: CAR T cell is a therapy attacks cancer cells after infusion into patients

A recent immunotherapy approach involves identifying T-cells with appropriate antitumor properties, then expanding them ex-vivo for treatment. Before infusion of the modified T-cells, the patient is lymphodepleted. This is because hematopoietic stem cells improve the expansion and mission of adaptively transferred anti-tumor CD8^+^ T-cells. The two types of ACT are CAR- T cells and TIL. CAR-T cells express a chimeric antigen receptor consisting of an extracellular immunoglobulin domain for antigen specificity and an intracellular T-cell activating domain. CD19-specific CAR-T cells are a successful example, now considered the gold standard for treating B-cell malignancies. TILs, in contrast, are selected based on CD137 expression, reflecting enhanced tumor reactivity and IFN production. Some studies have shown that zinc finger nucleases can be used to reduce PD-1 expression on TILs, resulting in enhanced effector function and chemokine production. CAR-T therapy has an advantage over TIL therapy in that CAR-T cells are not MHC-restricted and therefore cause less severe side effects than TILs. The reason behind that, is that MHC is expressed on both normal cells and tumor cells. to achieve the most optimum setting for ACT could be manipulated using ICI therapy to avoid TME limitations. Another challenge facing these advanced therapy medicinal products (ATMPs) is commercialization and large-scale manufacturing (Fig. 7) [106].

#### Chemotherapy and adjunctive strategies

Different chemotherapeutic agents were tested in combination with ICIs for targeting of MDSCs. For example, the most well-known nucleoside analog gemcitabine was studied with ICIs for treatment of different malignancies like mesothelioma. Gemcitabine was tested with oral checkpoint kinase I (CHK1 or SRA 737) for lung tumor. This combination was found to decrease the activity of MDSCs, PD-1 and Treg while improving macrophage activities [107].

Another example is the synergistic efficacy of the pyrimidine analogue 5 Fluorouracil (5-FU) with anti-PD1. This combination produced greater survival benefits than administration of 5-FU alone, as the single-agent 5-FU was found to enhance MSDCs efficacy and tumor angiogenesis [108–110]. Nap-paclitaxel, an albumin-bound formulation of paclitaxel, has been used in combination with atezolizumab for treatment of triple negative breast cancer with an immunosuppressive TME with evidence for high efficacy and safety [111]**.**

#### TGF-beta and Bintrafusp-alfa

Transforming growth factor β (TGF-β) is a good target for cancer treatment.. Several studies have shown that TGF-β inhibition is an effective way to improve T-cell infiltration into the tumor [22]. TGF-β plays an important role for regulation of different immune cells. It regulates adaptive immunity as well as the activity of natural killer cells, macrophages, and neutrophils. and it suppresses the function of effector T cells and antigen-presenting dendritic cells [112, 113]. TGF-β also plays a major role in development, homeostasis, and tissues renewal. Defects in TGF-β function can therefore contribute to immune abnormalities, fibrotic disorders, congenital defects, or malignancy. In premalignant cells, TGF-β acts as a potent tumor suppressor by activating cell-death programs. Once this function is mutated or suppressed, however, malignant cells instead exploit TGF-β to drive the differentiation and growth of further, using TGF-β itself to build an immunosuppressive TME, reinforcing stromal protection for the tumor, promoting tumor development, and encouraging metastasis [114]**.**

TGF-β belongs to a superfamily of ligands comprising 32 members, grouped into two main subfamilies, TGF-β and bone morphogenetic protein (BMP), based on their similarity in sequence and function [115]. The TGF-β subfamily includes ligands such as TGF-β1, TGF-β2, and TGF-β3, along with growth and differentiation factors such as GDF1, GDF3, and GDF8 (termed myostatin). TGF-β1 is the most immunologically relevant ligand for immune function [116]. Malignant cells exploit the TME to mobilize active TGF-β1, evading immune detection through expansion of the tolerogenic Treg compartment [117–119]**.**

The principle function of TGF-β is tumor repression through induction of apoptosis and inhibition of proliferation of malignant and pre-malignant cells. Under selective pressure, however, malignant cells deactivate the TGF-β signaling pathway and alter its function under. Malignant cells subsequently exploit TGF-β to drive tumor growth and metastatic behavior, allowing malignant cells to establish an immunosuppressive TME [115]. According to the most recent of human TCGA (The Cancer Genome Atlas) data, most malignancies, including lung squamous carcinoma, gastric carcinoma, colorectal carcinoma, pancreatic carcinoma, bladder, cervical, endometrial and head and neck carcinoma, exhibit TGF-β pathway alterations and mutations in at least 10% of reported cases involving TGF-β and tumor progression [114]. Therefore, interfering with TGF-β signaling pathway represents a challenging target for immunotherapies. However, the development of TGF-β based immunotherapies has progressed slowly. This slow progression is due mainly to two main factors. First, targeting of TGF-β may improve the patient survival or may alter the case into worse scenario relying on the role of TGF-β in TME. Second, the first generation of TGF-β inhibitor revealed severe cardiac toxicity. Nevertheless, several studies have examined drugs and techniques for targeting TGF-β as a promising approach to tumor suppression and metastasis reduction [114, 120].

Galunisertib (LY21577299) and Bintrafusp alfa are the most widely used agents targeting of TGF-β signaling pathway. Galunisertib is a small molecule that interfere with TGF-βR1 kinase function to inhibit the TGF-β pathway, blocking all three TGF-β ligands (TGF-β1,2,3). It activates T-cell infiltration and improves the effect of checkpoint therapy in animal model of colorectal carcinoma [121, 122]. Galunisertib is utilized in combination with checkpoint inhibitors nivolumab and durvalumab for treatment of head and neck cancer, and non-small cell lung carcinoma; the results of these clinical trials is still under investigation [123]. Galunisertib belongs to a well-known class termed as small-molecules inhibitors (SMIs), which are designed to block TGF-β I & II kinase receptors by inhibiting serine/threonine kinase A large panel of SMIs was screened by Eli Lilly for their ability to inhibit TGF-β kinase receptors using a TGF-β-dependent cell-based assay.

Galunisertib exhibited lower cardiovascular toxicity in animal models than the other small molecules inhibitors of kinase (SMIKs) group members. It also reduces the risk of developing of chronic inflammation in skin and gut, a well-established complication of TGF-β pathway blockade. Treatment with Galunisertib also helps overcome resistance of malignant cells to chemotherapeutic agents. Studies of TGF-β signaling biology have shown that the chemoresistance depends on the TME. This pathway includes over expression for TGF-β ligands, loss of MED12 gene, and resistance to epidermal growth factor receptor (EGFR). Treatment with Galunisertib, however, reduces these resistance pathways, even in MED12-deficient malignant cells [122]**.**

Bintrafusp alpha, in contrast, is a novel immunotherapy engineered as TGF-β trap with dual activity against as programmed cell death ligand (PDL1) and TGF-β receptor II, combining two moieties: immunoglobulin G (IgG1) moiety targeting PD-L1 and an extracellular TGF-β receptor II domain that blocks TGF-β, joined by a flexible peptide linker. The TGF-β receptor II moiety blocks the activity of the TGF-β1, −2, and −3 ligands, while the IgG1 moiety binds PD-L1. Bintrafusp alfa has shown strong efficacy in suppressing epithelial-mesenchymal transition (EMT) mediated by TGF-β. EMT is a reversible phenotypic shift in which malignant cells transition from an epithelial state with extensive cell–cell adhesion to a mesenchymal state with scattered, less adhesive, spindle-shaped cells with reduced or absent polarity, a shift that increases the ability of malignant cells to invade, metastasize, and resist radiotherapy and chemotherapy. This shift is primarily controlled by TGF-β [124, 125]. Bintrafusp also restrains TGF-β activity related to natural killer (NK) cells; TGF-β has been found to modify the NK-cell phenotype and suppress its cytotoxic and antitumor activity. The anti-PD-L1 moiety, meanwhile, activates antibody-dependent cellular cytotoxicity via NK cells in malignancies such as urothelial, cervical, and breast cancer [126–128]. Bintrafusp alfa also improves NK-cell infiltration into the TME and NK-cell activation, and promotes antitumor immune responses through its bifunctional PD-L1/TGF-β blockade. The dual mechanism of bintrafusp alfa also promotes T-cell proliferation, regulates Treg differentiation, and supports the T-cell compartment and antitumor response [129, 130]. Phase I clinical trials of Bintrafusp alpha have been completed, showing promising efficacy in patients with locally advanced or metastatic solid tumors, with or without concurrent checkpoint inhibitor therapy [131]. Several further trials are underway combining Bintrafusp alfa with other immunotherapies, chemotherapies, and radiation therapy [132].

#### TME modulators and Vascular Endothelial Growth Factor (VEGF) inhibitor antibodies

T-lymphocyte entry into the tumor begins with invasion of the tumor circulation, adherence to vascular endothelial cells, and finally migration across the vessel wall. Tumor vasculature therefore has a strong influence on T-cell infiltration into the tumor bed and TME [24]. This process depends on molecules such as intracellular adhesion molecules (ICAMs), P- and E-selectin, and vascular cell adhesion molecules (VCAMs) and vascular endothelial adhesion molecules [86]. Effective T-cell infiltration into tumor sites is often limited, however, by factors such as insufficient accumulation of adhesion molecules on tumor endothelial cells. Endothelin binding to the endothelin B receptor (ETBR) on the endothelial wall also reduces T-cell infiltration by lowering ICAM production. Meanwhile, tumors produce vascular endothelial growth factor (VEGF), which stimulates new vessel formation, increases vascular permeability, and reduces expression of adhesion molecules such as VCAM-1 [88, 133]. VEGF further enables Interleukin 10 (IL-10) and prostaglandin E2 (PGE2) to upregulate Fas ligand (FasL) pathway in tumor endothelial cells, eliminating tumor associated T-cells [134]. Inhibiting VEGF activity can therefore improve T-cell infiltration into the TME and overall prognosis.

VEGF is a principal driver of tumor angiogenesis, initiating the growth of endothelial cells. Angiogenesis is a complex process of forming new blood supply and plays an important role in different diseases such as psoriasis, arthritis, diabetes, as swell as malignancy. Most tumors rely on angiogenesis for substantial growth and metastasis. Tumor angiogenesis is leaky, irregular, and heterogenous, characterized by a lack of clear distinction between arterioles and venules, which facilitates tumor growth and metastasis. Tumor angiogenesis is influenced by various factors, including cytokines, tumor type, and proangiogenic genes; these factors can shift the tumor from a dormant to an invasive state, a transition termed the angiogenic switch [135]. Therefore, blocking of tumor angiogenesis and preventing tumor endothelial cell activity is therefore a promising strategy for halting tumor progression, offering an alternative to traditional chemotherapeutic agents [136]. Different mechanisms and approaches can be used to block tumor angiogenesis, such as binding angiogenic factors such as VEGF directly, blocking angiogenic receptors with anti VEGF- receptor antibodies, or using tyrosine kinase inhibitors to disturb intracellular signaling pathways [135]. Antiangiogenic treatment corrects the vascular abnormalities of malignancies, enhances immune cells infiltration, and improves tissue perfusion. Bevacizumab, the first U. S. Food and Drug Administration (FDA) approved angiogenesis inhibitor, was used in combination with atezolizumab in patients of with metastatic renal cancer and showed improved lymphocyte infiltration [137].

Bevacizumab (Avastin^®^) is a humanized anti-VEGF monoclonal IgG1 antibody that was approved for the treatment of advanced colorectal cancer, metastatic breast cancer, and non-small cell lung carcinoma in combination with chemotherapy, and can be used alone for glioblastoma as a second-line treatment, per approval by FDA (2010) and the European Medicines Agency (2008). Its main mechanism of action relies on the selective binding to circulating VEGF and inhibiting VEGF cell surface receptor engagement. This binding causes drooping tumor vascular growth, reduction of tissue interstitial pressure, elevation of vessels permeability and improve apoptosis of tumor endothelial cells [138]. Animal studies in mice have supported this mechanism: anti-VEGF therapy (bevacizumab) increased the sensitivity of tumor vasculature and slowed its growth to baseline rate within 7 days [139]. Avastin or Bevacizumab is a clear, colorless liquid administrated intravenously in combination with other chemotherapeutic agent at doses ranging from 5 to 15 mg/kg, with a maximum tolerated dose of 20 mg/kg at which roughly a quarter of patients experience headache, nausea, and vomiting as grade 3 toxicity of [140]. Bevacizumab can be detected in human serum using Enzyme-linked immunosorbent assay (ELISA) assay. Serum concentration analysis across patients receiving standard doses of 1 to 20 mg/kg revealed a half-life ranging from 11 to 50, averaging 19.9, with steady state reached by approximately 100 days [141]. This monoclonal antibody has a high protein binding (over 97%) to platelet-derived serum VEGF. This binding supports drug release at the endothelial site and targets tumor cell VEGF, which prevents tumor angiogenesis. This high binding also contributes to severe side effects such as gastrointestinal bleeding, hypertension, and impaired wound healing [140].

Pharmacokinetics study of bevacizumab exhibited that this drug follows two-compartment open model, with a volume of distribution (Vd) of 2.39 in women and 3.29 L in men. This Vd is close to plasma volume, consistent with primarily vascular distribution. Scintigraphic visualization of radiolabeled bevacizumab in mouse models showed greater accumulation in tumor tissue than in normal tissue [142, 143]. Bevacizumab undergoes cellular uptake by pinocytosis and binds the neonatal Fc receptor (FcRn) which protects it from systemic elimination and delays degradation [144]. VEGF gene showed a genetic variation and polymorphism at five functional single nucleotides. This variation can alter VEGF receptor expression, which may reduce treatment efficacy or increase and difference in patient responses [145]. As with most chemotherapeutic agents, tumor resistance to bevacizumab can develop through emergence of alternative angiogenic pathways, upregulation of VEGF receptors, and rapid tumor regrowth [139, 146]. Therefore, Administration bevacizumab in combination with other chemotherapeutic agents can therefore improve efficacy and reduce resistance. Pembrolizumab is another monoclonal antibody that acts through a distinct mechanism for the treatment of malignancies.

Pembrolizumab and nivolumab are anti-programmed cell death protein 1 monoclonal antibodies (anti-PD-1 antibodies) recently approved by FDA. The main difference between these two agents is that pembrolizumab is a fully humanized IgG4-kappa isotype, while nivolumab is a fully human IgG4 antibody [147]. Whereas, PD-1 is an immune checkpoint receptor with two ligands (PD-L1 and PD-L2) found mainly on activated immune cells such as B cells, T cells, and natural killer cells [148]. PD-L1 is expressed mainly on macrophages and shows up- and down-regulation in response to inflammatory signals [149, 150].

PDL2, in contrast, is found in macrophages or dendritic cells [151, 152]. Pembrolizumab or Keytruda, has shown checkpoint-inhibitory and antitumor activity. It binds PD-1 to block its engagement with its ligand, activating T-cell–mediated immunity against malignancies. Pembrolizumab and nivolumab were approved in 2014 for the treatment of malignancies such as melanoma and non-small cell lung cancer. In 2018, a third agent in this class, cemiplimab, was approved in the USA for the treatment of locally advanced and metastatic cases of cutaneous squamous cell carcinoma [153, 154]. Identification of the co-crystal structure of PD-1/PD-L1 and PD-1/PD-L2 was an important advance, revealing a clear picture of the interaction between these receptors and the monoclonal antibodies described above. These drugs belong to the IgG4 subclass, and their selective binding depends on the degree of interaction with the flexible loop of PD-1. Both pembrolizumab and nivolumab can effectively block the interaction of PD-1 with PD-L1, although they may in some cases show overlapping binding sites without affecting their inhibitory activity [155, 156].

#### Targeted hypoxia

Malignant tissues exhibit high metabolic rates and a correspondingly high demand for nutrients and oxygen, producing hypoxia. Hypoxia mainly arises from high oxygen consumption by rapidly dividing malignant cells combined with uneven oxygen feeding by tumor angiogenesis [157]. This hypoxia is the key element in angiogenesis process of tumor tissues. Hypoxia stimulates hypoxia inducible factor (HIF) that activates VEGF gene and induces transcription of VEGF protein, which binds VEGF receptors VEGF1 and VEGF2 and coreceptors neurolipin NRP-1 and NRP-2 [158, 159]. VEGF induces the proliferation of endothelial cells and formation of new vessels, resulting in impaired tissue perfusion and increasingly leaky vasculatures [88]. Thereafter, tumor vasculature exhibits great effect on T-cell infiltration in the tumor bed since T-cell entry requires invasion of the circulation and adherence to the endothelial cells [24]. T-cell lymphocytes reveal its activity by help of different molecules, including vascular cell adhesion molecules (VCAMs), intracellular adhesion molecules (ICAMs) and P- and E- selectin [86]. VEGF can reduce the expression of the previous molecules, particularly VCAM-1, on tumor endothelial cells, leading to lower T-cell infiltration [88]. Different studies showed that the higher leaky vasculatures in presence of impaired vascular tight junctions worsen hypoxia, necrosis, and acidosis in the TME, further reducing T-cells infiltration and affect antitumor immunity [86].

Hypoxia promotes the cold tumor phenotype through several mechanisms. It activates immunosuppressive cells and increases their accumulation within the TME. It also induces VEGF-driven angiogenesis, which reduces T-cell migration into the TME. Hypoxia also controls expression of CD39 and CD37 ectonucleotidases and transforming growth factor TGF-β [86, 160]. CD37 and 39 prevent the production of cytokines and proliferation of T-cells through catalyzing of the conversion of ATP to extracellular adenosine (ADO). Animal studies confirmed this: inhibiting ADO accumulation increases T-cell infiltration, improves antitumor immunity, and enhances natural killer (NK) cell function, which controls the malignancies [161, 162]. In different models and studies, hypoxia causes repression of NK cells, a population of cytotoxic lymphocytes that target malignant cells [163]. This repression is mediated by HIF upregulation and activation of PI3K/mTOR pathway [164]. Also, hypoxia may cause repression for the activity of natural killer cell receptors such as NK p30, NK p44, NK p44 and natural killer group 2D (NKG2D), impairing their expression and their capacity for tumor cell killing. Hypoxia also promotes regulatory T-cell (Treg) infiltration, which induces the immunosuppressive cytokine TGF-β to prohibit the activities of natural killer cell function [165, 166].

Hypoxia therefore plays a central role in both cancer progression and immune suppression. Making it a promising target for cancer immunotherapy is a potential concern for malignant fighting. Different strategies are under investigation for targeting hypoxia in cancer such as inhibition of HIF effect, metabolic interception, hypoxia activated prodrug (HAPs), downregulate of some hypoxic signaling such as mechanistic (mammalian) target of rapamycin (mTOR) and unfolded protein response (UPR) [167]. Formerly, it was mentioned that HIF plays an important role in hypoxic TME conditions across cancer cell lines. Targeting and inhibiting HIF function in combination with immunotherapy and chemotherapy is therefore a promising approach for treating malignancies. Drugs targeting the HIF pathway are classified into categories based on mechanism of action, including modulators that interfere with HIF dimerization, messenger RNA mRNA or protein expression and degradation, and DNA binding and transcriptional activity. CRLX101 is an example of this group, acting as a dual inhibitor of HIF-1α and topoisomerase-1; in combination with immunotherapy in preclinical models to get synergistic effect [168–170]. Hypoxia activated prodrugs (HAPs), or bioreductive prodrugs, are a class of biologically inactive compounds that become activated through enzymatic reduction, killing tumor cells within hypoxic regions of the TME [171]. TH-302 or evofosfamide is the one of the most successful [172] paradigms for this group and has been used with immunotherapy such as PD-1 blockade for the treatment of malignancies, showing a promising survival benefit in mouse prostate cancer models. Its known mechanism relies on its ability to direct T cells to the hypoxic TME and repression of myeloid stroma inside the tumor [157, 172]. Praziquantel is another example for this category. It is a mitomycin C derivative prodrug that is utilized for treatment of superficial bladder cancer [173]. As a complementary approach, oxygen is used as co-adjuvant therapy to reduce tumor hypoxia. Respiratory hyperoxia has been reported to improve pulmonary tumor control by approximately 60%, and to improve intra-tumoral T-cells infiltration [174, 175]. Also, Scharping group studies revealed that utilizing oxygen with metformin corrected hypoxic TME conditions, improved T-lymphocyte activity, and enhanced the enhanced the efficacy of PD-1 immunotherapy [176]. Therefore, this technique is an excellent way for fighting hypoxic TME conditions in the treatment of malignancies.

Specific treatment strategies also exist for tumors expressing low levels of surface antigen. Demethylating agents can restore tumor antigen expression. These agents have the ability to reverse chemokine silencing in the tumor bed, which otherwise negatively affects CD8^+^ promotion and patient outcome [177]. Other therapies include TGF B antagonists. TGF B promotes tumor proliferation, metastases, angiogenesis, immune and drug resistance [121]. One of the most common TGF B antibodies is PD 1 antibody, which promotes tumor regression [22]. Antiangiogenic drugs are another example, helping normalize tumor vasculature and enhance the adhesion of leukocytes to tumor endothelial cells. such drugs are used in synergy with ICI [22].

In summary, numerous emerging therapies remain under active investigation. Although many are not effective as monotherapies, several may allow for reduced chemotherapy dosing and correspondingly lower toxicity. Converting cold tumors into hot tumors will likely require pre-treatment to make them vulnerable to immune infiltration [178].

#### Immune suppressive cells in the tumor microenvironment

Myeloid-derived suppressor cells (MDSCs) is a heterogenous population of myeloid cells that represent a key component of the immunosuppressive TME. They affect the performance of immunotherapies and strategies through direct suppression of T lymphocytes and by establishing an immunosuppressive network that helps to mitigate the antitumor response. MDSCs modulate the TME through several mechanisms, including induction of hypoxia and tumor angiogenesis, and upregulation of immune checkpoints. Recruitment of immunosuppressive cells and activation of MDSCs therefore represent promising targets for overcoming resistance to ICIs and potentiating immunotherapy [179]. MDSCs accumulate in cancer patients within tumor sites, draining lymphoid tissues, and peripheral blood, where they establish the immunosuppressive network and attenuate T-lymphocyte cytotoxicity [180, 181]. MDSCs inhibits T-lymphocyte function through direct cell-to-cell contact or through specific mechanisms involving various mediators and receptors. For example, MDSCs may show elevated expression of arginase I (ARG I), which results in cell-cycle arrest and dysfunction of T lymphocytes through downregulation of T-cell receptor (TCR) α-chain expression [182, 183]. TCR signaling can also be attenuated and deactivated by MDSC-derived reactive oxygen species (ROS) and peroxy-nitrite, leading to impairment of CD8^+^ T lymphocytes. Furthermore, MDSCs may block T-cell activity by depleting cysteine through overexpression of the Xc − transporter [184, 185]. MDSCs mainly exhibit a strong relationship with cancer immune modification, and myeloid cells have also shown un expected pro-angiogenic effect for angiogenesis of malignancy [186]. The TME depends on extensive communication between endothelial, stromal, immune, and malignant cells to establish a powerful immunosuppressive network that supports cancer survival and growth. MDSCs represent a key link between these cell types, generating a positive feedback loop between immune and non-immune cells. They also act as an amplifier of this mechanism, boosting tumorigenic activity and promote resistance to ICIs [179]. Therefore, targeting of MDSCs represents a good strategy for converting the cold tumor into hot tumor.

Despite significant progress, conventional treatment strategies alone remain insufficient to fully reverse immune suppression in cold tumors. Recent advances in nanotechnology have opened new opportunities to target the tumor microenvironment more precisely, paving the way for innovative immuno-nanomedicine approaches.

## Nanotechnology as a promising approach

### Advantages of nanotechnology for cold and hot tumors

The limited responsiveness of immunologically “cold” tumors to conventional immunotherapies has emerged as a major obstacle in oncology, primarily due to their low immunogenicity, poor antigen presentation, and restricted infiltration of effector immune cells. These tumors are typically characterized by an immunosuppressive tumor microenvironment (TME), deficient in pro-inflammatory signals and enriched with stromal and metabolic barriers that collectively hinder effective antitumor immunity [9, 41]. Consequently, there is a growing need for innovative therapeutic platforms capable of reprogramming the TME and converting cold tumors into immunologically active “hot” tumors.

Nanotechnology has recently gained significant attention as a transformative approach in this context, offering unique physicochemical and functional properties that enable precise modulation of tumor biology at multiple levels. Unlike conventional therapies, nanocarriers can be rationally engineered to achieve targeted delivery, controlled release, and spatiotemporal activation within the tumor milieu, thereby overcoming biological barriers associated with cold tumors [187, 188]. Importantly, nanoplatforms can be designed to co-deliver immunomodulatory agents, tumor antigens, and adjuvants, facilitating the initiation and amplification of antitumor immune responses.

A key advantage of nanotechnology lies in its ability to remodel the tumor microenvironment by addressing hallmark features of cold tumors, including hypoxia, acidosis, and immune exclusion. For instance, nanoparticles can be utilized to normalize abnormal tumor vasculature, enhance immune cell trafficking, and promote dendritic cell maturation and antigen cross-presentation, thereby restoring critical steps of the cancer-immunity cycle [189]. Furthermore, emerging nanomaterials have demonstrated the capacity to induce immunogenic cell death (ICD), a process that enhances tumor antigen release and stimulates adaptive immune responses, which is particularly beneficial in poorly immunogenic tumors [190, 191].

In addition to their immunomodulatory functions, nanotechnology-based systems enable the integration of diagnostic and therapeutic modalities within a single platform (theranostics), allowing real-time monitoring of treatment response and personalized therapeutic interventions. This is particularly relevant in cold tumors, where treatment efficacy is often unpredictable and requires dynamic assessment [192]. Collectively, these advances position nanotechnology as a pivotal tool in next-generation cancer therapy, offering promising strategies to overcome immune resistance and enhance the clinical outcomes of patients with cold tumors.

Nanomedicines are now used in combination with immunotherapy for the treatment of malignancies and to convert cold tumor into hot tumor. Three levels represent the main target of the nanomedicine: the tumor microenvironment, tumor cells, and the peripheral immune system, which can be reached through two different approaches, of passive and active targeting [193, 194]. Passive targeting relies on the enhanced permeability and retention (EPR) effect to deliver nanomedicine to the tumor, while active targeting relies on ligands directed at specific tumor- expressed receptors, such as transferrin and peptides [195, 196]. Various strategies have been used to disrupt the immunosuppressive TME and slow tumor progression, including immune checkpoint inhibitors (ICIs), cancer vaccines, and adoptively transferred antigen-specific T-lymphocytes. ICIs are widely used to reactivate the response of endogenous T-cells. This approach has been successfully applied to malignancies such as head and neck carcinoma, breast cancer, metastatic melanoma, liver cancer, renal cancer, and lung and colorectal cancer. Promising findings have emerged from previous clinical trials; however, therapeutic resistance to ICIs limits the broader application of this strategy [197, 198].

Nanomedicines targeting tumor cells work by improving the tumor immune state and inducing immunogenic cell death (ICD). Doxil is a representative example of this category, consisting of a PEGylated liposomal formulation of the well-known chemotherapeutic agent doxorubicin. Doxil induces T-cell proliferation and infiltration of tumor cells via ICD and shows greater activity than free doxorubicin [193, 199, 200]. Nanomedicine-based targeting of the TME, in contrast, relies on inhibiting immunosuppressive cells such as tumor-associated macrophages (TAMs) and immunosuppressive molecules such as adenosine and TGF-β, or on augmenting the function of immune cells such as macrophages [193, 201]. Finally, the peripheral immune system-targeting nanomedicine relies on promoting antigen presentation and cytotoxic T-lymphocytes (CTL) generation in draining lymph nodes. For example, nanomedicine-based vaccines enhance antigen presentation, improve the level of CTL activation, and promote antigen delivery to lymph nodes [193, 202, 203].

Focused ultrasound, magnetic hyperthermia, and tumor phototherapy are well-known examples of nanotechnology applications that target tumor cells. Tumor phototherapy includes two main branches: photothermal therapy (PTT) and photodynamic therapy (PDT) which is commonly used for superficial solid tumors. PTT depends on a near-infrared light source that is absorbed by a photothermal agent such as graphene oxide or organic nanoparticles. The nanoparticles convert the infrared laser energy into thermal heat that destroys tumor cells. PDT, in contrast, relies on the delivery of photosensitizers that can be activated by laser light to damage the nucleus and DNA of tumor cells. Both PTT and PDT also improve immunotherapeutic responses and CTL infiltration, since the chemical and thermal cell damage they cause activates immunogenic cell death (ICD) [196]. It is preferred to use the both techniques together or in a combination with immunotherapy as the individual treatment has limited effect due to the weak penetration power of light [204].

Magnetic hyperthermia is a technique that relies on magnetic nanoparticles in the presence of a magnetic field, converting magnetic energy into thermal energy that causes direct cell death and induces ICD. This technique offers advantages over phototherapy due to greater tissue penetration, more precise targeting, and better control. For example, iron oxide nano-rings used to treat breast tumors have shown significant synergy with antiangiogenic treatments such as anti-PD-L1 antibodies [205, 206].

High-intensity focused ultrasound (HIFU) represents one of the recent techniques that is used for the diagnosis and treatment of malignancies. It is an entirely non-invasive technique that has shown strong effects against solid and metastatic tumors. This technique relies on transferring a sufficient quantity of energy to raise cellular temperature to a lethal level. This heat coagulation causes tissue damage and fat necrosis in the surrounding normal fatty tissue [207]. Several studies have shown that HIFU has lower toxicity compared with other invasive techniques such as laparoscopic radiofrequency ablation, cryotherapy, and percutaneous alcohol ablation. These techniques also have an upper tumor size limit of 3 to 4 cm during application [208]. Some experimental studies suggested that HIFU may improve human immune response against tumors through mechanisms that remain unclear. For example, the application of HIFU in a rabbit VX2 bone tumor model activated T-lymphocyte proliferation and an antitumor immune response. A subsequent clinical trial in 16 patients with solid malignancies revealed increases in CD4 and CD8 cell counts and a significant reduction in circulating tumor cells after HIFU treatment. It was therefore hypothesized that the HIFU technique induces extensive tumor necrosis and excess tumor antigen release, which improves immune function in patients and leads to better prognosis [209, 210].

Targeting the peripheral immune system represents a promising way to engage the TME and tumor cells, for example by targeting antigen-presenting cells (APCs) in secondary lymphoid organs and T cells in the circulation. Antigens can also be transferred to the peripheral immune cells and organs via cancer vaccination using nanocarriers such as ionizable lipid nanoparticles carrying mRNA that encodes patient-specific antigens [211]. Another example for peripheral immune system targeting involves loading a leukemia-specific 19−41BBζ gene into a nanocarrier to drive expression of chimeric antigen receptors, an alternative approach to conventional CAR-T therapy [212].

### Recent strategies and nanoplatforms for treatment of cold tumors

Inorganic nanoparticles have recently emerged as a promising drug delivery platform for the treatment of cancer [213, 214]. Calcium based nanoparticles represents one of the most preferred nanoplatforms in cancer treatment today. They are biocompatible carriers with high efficiency as a drug carrier [215, 216]. The ability of modulation of calcium nanoparticles by various functional nanomaterials allows them to act through different mechanisms of cancer cell killing, such as induction of apoptosis [217]. It shows several advantages like its ability to work effectively in the absence of ultrasound, light, or heat as external stimuli [218, 219]. Also, the nanoparticles can also be produced from inexpensive, biocompatible, and biosafe raw materials [220]. Calcium-based nanoparticles are classified into five categories, of which the most common is calcium carbonate NPs [221]. The most important feature of calcium carbonate NPs, its robust buffering capacity, which allows them to modulate tumor immunity by alleviating the acidic tumor microenvironment [222]. Furthermore, it plays as an ideal carrier for protein, genetic material, and ultrasound imaging-guided therapy, with minimal side effects, since calcium and CO_2_, their main metabolic byproducts, can be cleared through bone accumulation, renal excretion, and pulmonary exhalation [222–224]. Several studies have demonstrated the efficacy of calcium carbonate nanoparticles in tumor treatment. For example, Zhao et al. (2022) showed their ability to cross the blood–brain barrier (BBB), inducing a necrosis-driven immune response in the treatment of glioblastoma [225].

As noted earlier, the immunosuppressive tumor microenvironment (TME) is responsible for limiting immunotherapy by restricting infiltration of antitumor immune cells [226, 227]. Various pro-inflammatory strategies mediated by cytokines or immune checkpoint inhibitors have been proposed to enhance tumor-resident immune cells and cancer immunotherapy. Unfortunately, these strategies carry several side effects and limitations, including the pleiotropic effects of cytokines, the narrow genetic applicability of ICIs, and limited clinical trial availability [228–231]. New treatment strategies are therefore essential. A deficiency of nutritional metal cations is commonly observed in cold tumor tissue. Supplementation with essential metal cations, termed metallo-immunotherapy, therefore, represents a promising cancer immunotherapeutic strategy [232, 233]. The essential divalent metals such as Ca^2^^+^, Mn^2^^+^, and Zn^2^^+^ play an active role in activating antitumor immune cells [234–236]. It exhibits several processes, including induction of immunogenic cell death (ICD), enhancement of the autophagy pathway and dendritic cell antigen cross-presentation, induction of antigen-specific immune responses, and maintenance of CD8^+^ cell cytotoxicity [237–243].

Combining the aluminum adjuvant (Alum) with magnesium hydroxide (Imject Alum), which induces a T helper 1 (Th1)-polarized immune response, represents another strategy for treating cold tumors. Aluminum hydroxide gel (Al-hydrogel-AlOOH), or Alum, is widely used in animal and human vaccines to promote antibody production, but it generally fails to enhance the antitumor T-cell response. Adding Mg(OH)_2_ to Alum, however, improves the Th1-polarized response. NanoAlum is a nanoscale formulation of this combination, structurally similar to aluminum layered double hydroxide, that has been used as a vaccine adjuvant. It improves the cytotoxic T-cell response against solid tumor [244].

### Translational evidence of nanotechnology strategies for reprogramming the tumor immune

The studies were summarized in Table 2 that demonstrates that nanotechnology-based platforms can effectively modulate the tumor immune microenvironment through multiple mechanisms, including induction of immunogenic cell death, enhancement of antigen presentation, inhibition of immunosuppressive cell populations such as MDSCs, and activation of innate immune pathways such as stimulator of interferon genes (STING) signaling. Collectively, these strategies promote T-cell infiltration and activation, thereby facilitating the conversion of immunologically cold tumors into hot, therapy-responsive phenotypes. In clinical settings, early-phase trials of nano-vaccines and nanoparticle-based immune checkpoint delivery systems further support the translational potential of these approaches.

**Table 2:** Summary of nanotechnology-based strategies for converting cold tumors into hot tumors and their underlying immunomodulatory mechanisms

Strategy/Platform	Model Type	Mechanism of Action	Immune Effect	Stage
Nanocarriers (liposomes, polymers) [245]	Preclinical tumor models	Improved drug delivery & tumor accumulation (EPR effect)	Enhanced antigen exposure, TME modulation	Preclinical
Physicochemical nanoparticle tuning [246]	Preclinical cancer models	Optimized size/shape/surface for immune interaction	Improved immune cell engagement	Preclinical
EPR-based tumor targeting [247]	Solid tumor models	Passive accumulation in tumor vasculature	Increased local immune activation potential	Preclinical
STING-agonist nanoparticles [248]	Murine tumor models	Activation of cGAS–STING pathway → Type I IFN	↑ CD8 ^+^ T-cell infiltration, dendritic activation	Preclinical
mRNA lipid nanoparticles (neoantigens) [249]	Preclinical cancer models	Antigen expression in APCs → T-cell priming	Strong adaptive immune activation	Preclinical
Personalized neoantigen vaccines [250]	Clinical melanoma trial	Neoantigen-driven dendritic cell activation	Robust tumor-specific T-cell response	Phase I Clinical

### Surgery and application of nanotechnology

Because surgery is the primary treatment for cancer, particularly in its early stages, the ability to identify the tumor mass intraoperatively is highly important. Various instruments are used in tumor surgery. In contrast to other imaging modalities, optical apparatus offers particular advantages for intraoperative cancer diagnosis. Optically labeled tumors are visible to the human eye within the visible spectrum [242, 251, 252] and can be removed by the surgeon alone. For instance, standard fiber optics and silicon-based charge-coupled device (CCD) cameras can be used for tumor visualization in the near-infrared band with good sensitivity and minimal expense. Optical approaches can also be used to concurrently detect two or more cancer biomarkers and provide information on both signal intensity and wavelength [45, 46]. This wavelength-multiplexing feature enables studies of in vivo tumor heterogeneity (variation from region to region within the same tumor or from one tumor to another) as well as multicolor ratiometric" cancer imaging. SERS nanoparticles, with their distinctive spectroscopic signatures, are particularly well suited to multiplexed tumor targeting and detection, since they are biocompatible and nontoxic and can be produced with consistent particle size, surface coating, and in vivo pharmacokinetic properties. Like semiconductor quantum dots (QDs), their multicolor fluorescence emission and broad absorption profiles make them suitable for spectral multiplexing [253, 254].

Several additional techniques have also been used to facilitate surgical tumor treatment. For example, fluorescence imaging relies on absorption of light by tissue from an array of light sources, followed by emission of a fraction of that light a few seconds later. The emitted light is captured mainly by a detector array. There are now hundreds or thousands of fluorescent probes that provide precise labeling for applications such as biological systems, small molecules, peptides, affibodies, and substrate- or activity-based imaging [255].

For most malignancies, radical surgical resection is the recommended course of action, driving continued interest in techniques that improve resection accuracy, along with techniques for visualizing and protecting surrounding tissue and nerves. For surgery to be more accurate and successful, information about the tumor and adjacent tissues is essential. It is therefore important to use recent advanced techniques such as imaging camera prototypes together with appropriate fluorescent dyes. Examples of intraoperative testing for ovarian, breast, cervical, and bladder cancers, as well as melanoma, include fluorescence cystoscopy, image-guided transoral robotic surgery, and multispectral normalized fluorescence imaging systems [256]. The real-time intraoperative cancer detection in the presence of fluorescent labeling remains under investigation. For glioblastoma, head and neck cancer, and pancreatic cancer, the anti-epidermal growth factor receptor conjugate cetuximab-IRDye800 (LI-COR Biosciences) has been investigated [256].

A further challenge is that increased tissue scattering and blood absorption limit the penetration depth of optical technologies. Penetrating tissue deeper than 1 cm remains difficult, even within the near-infrared clear window. However, since tumors are surgically exposed and can be illuminated and detected optically, this is no longer a major obstacle; a shallow penetration depth may even help the surgeon localize the tumor precisely within a complex surgical field [257, 258]. A further issue is that solid tumors tend to resist thorough penetration by nanoparticles and macromolecules such as monoclonal antibodies. However, since nanoparticles are identifiable at the tumor perimeter, deep penetration is not required for accurate identification of tumor margins during surgery [259].

Frangioni and colleagues developed an operable prototype specifically designed for use in large-animal surgery, which could inform future clinical systems. Using this prototype and the near-infrared dye indocyanine green (ICG). Image-guided cancer resection with real-time surgical margin assessment, sentinel lymph node mapping, intraoperative tumor and normal vasculature mapping, avoidance of critical structures such as nerves, and intraoperative detection of occult metastases are just a few of the cancer-specific applications of this imaging system. For large-animal surgery and visualization of fluorescent particles, a more sophisticated device, the FLARE system (fluorescence-assisted resection and exploration), has also been developed. The surgical field is illuminated by two light sources, one for white light and one for near-infrared fluorescence excitation. A motorized zoom lens collects the white light reflected from the surgical field and directs it to a color video camera. Near-infrared fluorescence emission from the surgical field is typically minimal. However, when an exogenous near-infrared fluorophore, such as a lymphatic tracer, is used, the near-infrared camera captures the fluorophore's otherwise imperceptible fluorescent light. To display "anatomy" (from the color video camera) and "function" (from the near-infrared camera) separately or in combination, both cameras capture their images simultaneously [260, 261].

Another method of visualizing tumors during surgery uses quantum dots (QDs) conjugated to antibodies, Gao et al. [262]. reported the first instance of simultaneous in vivo targeting and imaging of tumors in living animals [252]. In murine models, nanoparticle uptake at tumor sites was achieved through active prostate-specific antigen targeting as well as passively enhanced permeability and retention. For effective background reduction and accurate delineation of faint spectral signals, a whole-body macro-illumination system was combined with wavelength-resolved spectral imaging. This study demonstrated that QDs can accurately localize tumors, a capability that is essential for oncologic surgery. Delivering sufficient QDs to render a tumor visible, whether to the naked eye or with imaging instruments, is the essential first step for any intraoperative [262].

Nanotechnology for cancer imaging also includes several other techniques, such as imaging of tumor-associated receptors, enzyme activity, and glucose uptake and tissue pH. In receptor imaging, several recent studies have targeted folate receptors in breast cancer. Folate receptor alpha is a potential anticancer drug target due to its overexpression in solid tumors. In one study, 24 mice were injected with iron oxide nanoparticles to reveal heterogeneous expression of folate receptor alpha in breast tumors. This study was carried out using positive cytoplasmic and cytoplasmic membrane staining for folate receptor alpha, along with intracellular staining for iron [263, 264]. As an example, for imaging enzyme activities, cathepsin-responsive gold nanoparticles were developed by Tsvirkun et al. to recognize the sequences of up to four amino acids flanking the enzyme cleavage site. These nanoparticles became visible on CT imaging following elevated tumor cathepsin activity in breast cancer implants in mice [265]. Motiei et al. developed glucose-functionalized gold nanoparticles detectable by CT contrast imaging to address a key limitation of 18F-FDG PET, its inability to distinguish tumors from inflammatory processes, since both show increased glucose uptake. The enhanced permeability and retention (EPR) effect was the main driver of glucose-nanoparticle accumulation at the tumor site. Overexpression of glucose transporters (GLUTs), such as GLUT-1, on the tumor surface further contributed to clearer, tumor-localized CT signals [266]. The fabrication and formulation of additional nanoparticle-based imaging agents remains essential for improving in vitro and in-vivo detection and is important for both preclinical trials and surgical treatment of tumors. The toxicity, stability, and detection precision of nanoparticle-based imaging in preclinical animal models remain in translational challenges that must be addressed. A biocompatible, biodegradable nanoparticle that is inert to the biological system while carrying drug molecules, dyes, enzymes, or other targeting molecules that could facilitate the siege of the tumors, is a challenge that should be achieved as soon as possible.

## Conclusion and perspective

Cancer remains a leading global health concern, with a high incidence rate and standing as the second-leading cause of death worldwide. The hallmarks of cancer enhance its capacity for proliferation, survival, metastasis, and angiogenesis, while also helping cancer cells reduce oxygen dependence, suppress apoptosis, and promote invasion. This review has highlighted the critical role of the tumor microenvironment in determining the success or failure of cancer treatment, particularly immunotherapy, and has demonstrated the potential of combining nanomedicine with immunotherapy to alter the behavior of cold tumors. Finally, this review examined the integration of immunotherapy and nanotechnology-based therapies, which provide new opportunities for effective management of cold tumors. Future research should focus on optimizing nanocarrier design to achieve precise modulation of the tumor immune microenvironment and enhance immune cell infiltration. Advanced biomaterials and intelligent delivery systems may further support targeted activation of immune pathways in cold tumors. Combining nanotechnology with gene editing or personalized immunotherapy approaches also holds considerable promise for transforming current cancer treatment paradigms and improving long-term patient outcomes.

## Data Availability

No new data were generated or analyzed in this study. As this is a review article based exclusively on previously published literature, no datasets were deposited in a public repository, and no persistent identifiers (e.g., DOI or accession numbers) or dataset hyperlinks are available. All supporting information is contained within the cited references.

## Abbreviations

5-FU: 5-Fluorouracil
ACT: Adoptive T cell transplant
ADO: Adenosine
AKT: Protein kinase B
AlOOH: Aluminum hydroxide (Al-hydrogel)
APC: Antigen presenting cell
ARG1: Arginase I
ATMPs: Advanced Therapy Medicinal Products
BATF3: Basic leucine zipper transcription factor ATF-like 3
BAGE: B melanoma antigen
BCG: Bacillus Calmette–Guérin
BMP: Bone morphogenetic protein
CAF: Cancer-associated fibroblasts
CAR T: Chimeric Antigen Receptor
CCL: CC chemokine ligand
CD8-12: Cluster of differentiation 8–12
cDC1: Conventional dendritic cells 1
CGA: Cancer germline antigen
CHK I: Checkpoint kinase I
CK: Chemokines
CTL: Cytotoxic T-lymphocytes
CXCL: CXC chemokine ligand
DAMP: Danger Associated Molecular Patterns
DC: Dendritic cells
DNMTi: DNA methyltransferase inhibitors
ECM: Extracellular matrix
EGFR: Epidermal growth factor receptor
ELISA: Enzyme-linked immunosorbent assay
EMT: Epithelial mesenchymal transition
EPR: Enhanced permeation and retention (effect)
ERAP: Human endoplasmic reticulum aminopeptidase
ETBR: Endothelin B receptor
EZH2: Zeste homolog 2
FasL: Fas ligand
FcRn: Neonatal Fc receptor
FDA: U.S. Food and Drug Administration
FDG: Fluorodeoxyglucose
FRP1: Formyl Peptide Receptor 1
GAGE: G melanoma antigen
GLUTs: Glucose transporters
GM-CSF: Granulocyte-macrophage colony-stimulating factor
HA: Hyaluronic acid
HAP: Hypoxia activated prodrug
HIF: Hypoxia inducible factor
HIFU: High intensity focused ultrasound
hTERT: Human telomerase reverse transcriptase
ICAMs: Intracellular adhesion molecules
ICD: Immunogenic cell death
ICG: Infrared dye indocyanine green
ICI: Immune checkpoint inhibitors
ICP34.5: Infected cell protein 34.5 (HSV virulence gene)
ICP47: Infected cell protein 47
IDO: Indoleamine 2,3-dioxygenase
IgG: Immunoglobulin G
IFN-γ: Interferon γ
IL10: Interleukin 10
LC3: light-chain 3
M_1_-TAMs: Tumor associated macrophages
MAGE-1: Melanoma associated antigen 1
MAPK: Mitogen-activated protein kinase
MDSCs: Myeloid-derived suppressor cells
MHC: Major histocompatibility complex
mRNA: Messenger RNA
mTOR: Mechanistic (mammalian) target of rapamycin
NK: Natural killers
NKG2D: Natural killer group 2D (receptor)
NP/NPs: Nanoparticle(s)
NRP-1: Neurolipin
NSCLC: Non-small cell lung cancer
OVs: Oncolytic viruses
PAMPs: Pathogen-associated molecular patterns
PAP: Prostatic acid phosphatase
PDAC: Pancreatic ductal adenocarcinoma
PDL1: Programmed cell death ligand 1
PDT: Photodynamic therapy
PEG2: Prostaglandin
PEGPH20: Pegvorhyaluronidase
PI3K: Phosphatidylinositol 3-kinases
PRR: Pattern recognition receptors
PTD: Photothermal-dynamic (combined PTT/PDT context)
PTEN: Phosphatase and TENsin homolog deleted on chromosome 10
PTT: Photothermal therapy
QDs: Quantum dots
RAS: Reticular activating system
ROS: Reactive oxygen species
SIRPα: Signal regulatory protein α
SMIKs: Small molecule inhibitors of kinases
SMIs: Small molecule inhibitors
STING: Stimulator of interferon genes
TA: Tumor antigen
TAA: Tumor associated antigens
TAMs: Tumor associated macrophages
TCRs: T cell receptors
TGF-β: Transforming growth factor beta
TIL: Tumor-infiltrating lymphocytes
TLS: Tertiary lymphoid structure
TMB: Tumor mutational burden
TME: Tumor microenvironment
TP53: Tumor protein 53
Tregs: T regulatory cells
TSA: Tumor specific antigens
T-VEC: Talimogene laherparepve
UPR: Unfolded protein response
VCAM: Vascular cell adhesion molecule 1
VEGF: Vascular endothelial growth factor
